# Increased c-Fos immunoreactivity in anxiety-related brain regions following paroxetine discontinuation

**DOI:** 10.1016/j.neuropharm.2025.110541

**Published:** 2025-06-02

**Authors:** Helen M. Collins, L. Sophie Gullino, Cara Fuller, Raquel Pinacho, David M. Bannerman, Trevor Sharp

**Affiliations:** a Department of Pharmacology, University of Oxford, OX1 3QT, UK; b Department of Experimental Psychology, University of Oxford, OX2 6GG, UK

**Keywords:** SSRI, paroxetine, discontinuation, mice, c-Fos, immunohistochemistry, anxiety

## Abstract

Selective serotonin reuptake inhibitor (SSRI) therapy cessation often induces a disabling discontinuation syndrome, including increased anxiety. We recently reported that SSRI discontinuation induced behavioural changes in mice, which we hypothesise arose from activated anxiety circuitry. Here, we investigated the effect of discontinuation from the SSRI paroxetine on the expression of the activity-dependent gene *c-fos* in selected anxiety-related midbrain and forebrain regions. Male mice were injected daily with paroxetine (10 mg/kg) or saline for 12 days, then treatment was either continued or discontinued for two or five days. Mice were then tested on the elevated plus maze (EPM) and tissue collected 90 min later. Brain sections including the dorsal (DRN) and median raphe nucleus, periaqueductal grey, hippocampus, prefrontal cortex, and amygdala were processed for c-Fos immunoreactivity. Two days after paroxetine discontinuation, when mice showed elevated anxiety-like behaviour on the EPM, increased c-Fos immunoreactivity was evident in the DRN and ventral hippocampus, but not in any other region examined, compared to saline-treated controls. Increased c-Fos in the DRN was evident in TPH2-immunopositive neurons as well as neurons doubled-labelled for TPH2 and VGLUT3, suggesting activation of 5-HT-glutamate co-releasing neurons. Five days after paroxetine discontinuation, increased c-Fos immunoreactivity was evident in the DRN, but mice no longer exhibited increased anxiety. These findings suggest that, under these conditions, paroxetine discontinuation is associated with a short-lasting activation of anxiety-promoting circuitry limited to DRN 5-HT neurons and the hippocampus. This circuitry may contribute to symptoms such as anxiety that are a feature of SSRI discontinuation syndrome.

## 1 Introduction

Selective serotonin reuptake inhibitors (SSRIs) are the first-line pharmacological treatment for major depressive disorder and several anxiety disorders. However, abrupt cessation of SSRI therapy can produce a debilitating discontinuation syndrome, comprising anxiety, sleep disruption and sensory disturbances amongst other symptoms (Haddad, 1997; Fava et al., 2015). These symptoms appear within a few days of SSRI discontinuation and can last for several weeks, and possibly longer (Davies and Read, 2018; Davies and Read, 2019; Iacobucci, 2019; Henssler et al., 2024).

Early preclinical studies report various behavioural effects of SSRI discontinuation in rodents. For instance, Bosker et al. (2010) reported that citalopram discontinuation in rats increased the acoustic startle response compared to continued citalopram treatment, which is a hypervigilance state interpreted by the authors as evidence of increased anxiety. Moreover, we recently showed that discontinuation from citalopram or paroxetine increased anxiety-like behaviour on the elevated plus maze (EPM) in mice compared to continued paroxetine and saline controls (Collins et al., 2022). The latter behavioural change was detectable at two but not five days after discontinuation, suggesting a short-lasting effect of discontinuation on anxiety-like behaviour (Collins et al., 2024). Nonetheless, we also observed alterations in sleep structure and reduced weight gain that lasted over a week following paroxetine discontinuation (Collins et al., 2023). Taken together these behavioural findings in animals are reminiscent of some of the symptoms often experienced by patients undergoing SSRI discontinuation (Haddad, 1997). The neural pathways underlying the symptoms of SSRI discontinuation in animals or humans, however, are not known.

Previously, expression of the activity-dependent immediate early gene c-*fos* has been used in immunohistochemistry studies to reveal the neural circuitry activated by a variety of anxiety-generating settings and stimuli, including anxiety-inducing tasks and anxiogenic drugs. For example, exposing rats to various novel, anxiety-provoking environments increased the number of c-Fos immunoreactive neurons in the ventral hippocampus, paraventricular nucleus of the hypothalamus (PVN) and basolateral amygdala (BLA) (Boguszewski and Zagrodzka, 2005; Gomes et al., 2005; Muigg et al., 2009). Administration of anxiogenic drugs also evoked c-Fos expression in these regions and others including the prefrontal cortex (PFC) and periaqueductal grey (PAG), as well as the dorsal raphe nucleus (DRN) and median raphe nucleus (MRN) (Singewald and Sharp, 2000; Singewald et al., 2003; Netto and Guimarães, 2004), which are the main source of 5-HT innervation of the forebrain (Jacobs and Azmitia, 1992).

Findings such as these support the notion that there are multiple neural networks mediating anxiety-related behaviours, which include inputs from neuromodulatory systems such as 5-HT (Tovote et al., 2015). The 5-HT system is particularly relevant in the study of discontinuation from SSRIs given that these drugs target the 5-HT transporter. Interestingly, recent evidence suggests that SSRI discontinuation may be associated with 5-HT neuron activation; neurochemical studies showed that SSRI discontinuation evoked a rebound increase in tissue levels of 5-HT and its metabolite 5-HIAA in the hippocampus and other brain regions (Caccia et al., 1993; Trouvin et al., 1993; Stenfors and Ross, 2002; Bosker et al., 2010; Guiard et al., 2012; Collins et al., 2024). Indeed, we recently observed increased c-Fos immunoreactivity in the DRN of mice discontinued from paroxetine (Collins et al., 2024). However, the downstream effect of such 5-HT activation on anxiety-related brain structures is unclear.

Here, we investigated the effect of paroxetine discontinuation on c-Fos immunoreactivity in a range of forebrain and midbrain regions linked to anxiety circuitry. This included a detailed exploration of c-Fos immunoreactive neurons in subregions of the DRN and MRN, particularly those immunopositive for the 5-HT marker tryptophan hydroxylase2 (TPH2). We also analysed neurons that co-express TPH2 and the vesicular glutamate transporter, VGLUT3, and thereby co-release 5-HT and glutamate (Gras et al., 2002; Schäfer et al., 2002). The latter comprise an important subpopulation of midbrain 5-HT neurons that have recently been linked to anxiety-like behaviours and stress responses (Sengupta et al., 2017; Ren et al., 2018; Gullino et al., 2024).

## 2 Methods and Materials

### 2.1 Animals

C57BL/6J male mice (7 weeks, Charles River) were habituated to the holding facility for 1 week prior to experiments. Mice were group housed (3 per cage of littermates) at 21 °C on a 12 h light/dark cycle in open-top cages lined with sawdust bedding (plus cage enrichment consisting of sizzle nest, cardboard house, and tunnel) with *ad libitum* access to food and water. Only male mice were included as previous experiments did not detect evidence of increased anxiety in female mice following paroxetine discontinuation (Collins et al., 2022). Experiments followed ARRIVE guidelines and were conducted according to the UK Animals (Scientific Procedures) Act of 1986.

### 2.2 Drug treatment

Mice were allocated to one of the following groups (12 mice per group) by stratified randomisation: (i) saline; (ii) continued paroxetine; or (iii) paroxetine discontinuation. Mice received 10 mg/kg *s.c*. paroxetine (Abcam ab120069; 1 mg/ml in saline) or the equivalent volume of saline once-daily for 12 days, then treatment was either continued (saline and continued paroxetine groups) or swapped to saline injections (discontinuation group) for a further 2 or 5 days. These timepoints were chosen based on previous experiments showing that increased anxiety-like behaviour was evident on the EPM two days after paroxetine discontinuation, but not five days after (Collins et al., 2022; Collins et al., 2024).

### 2.3 Behavioural testing

**Figure 1:**
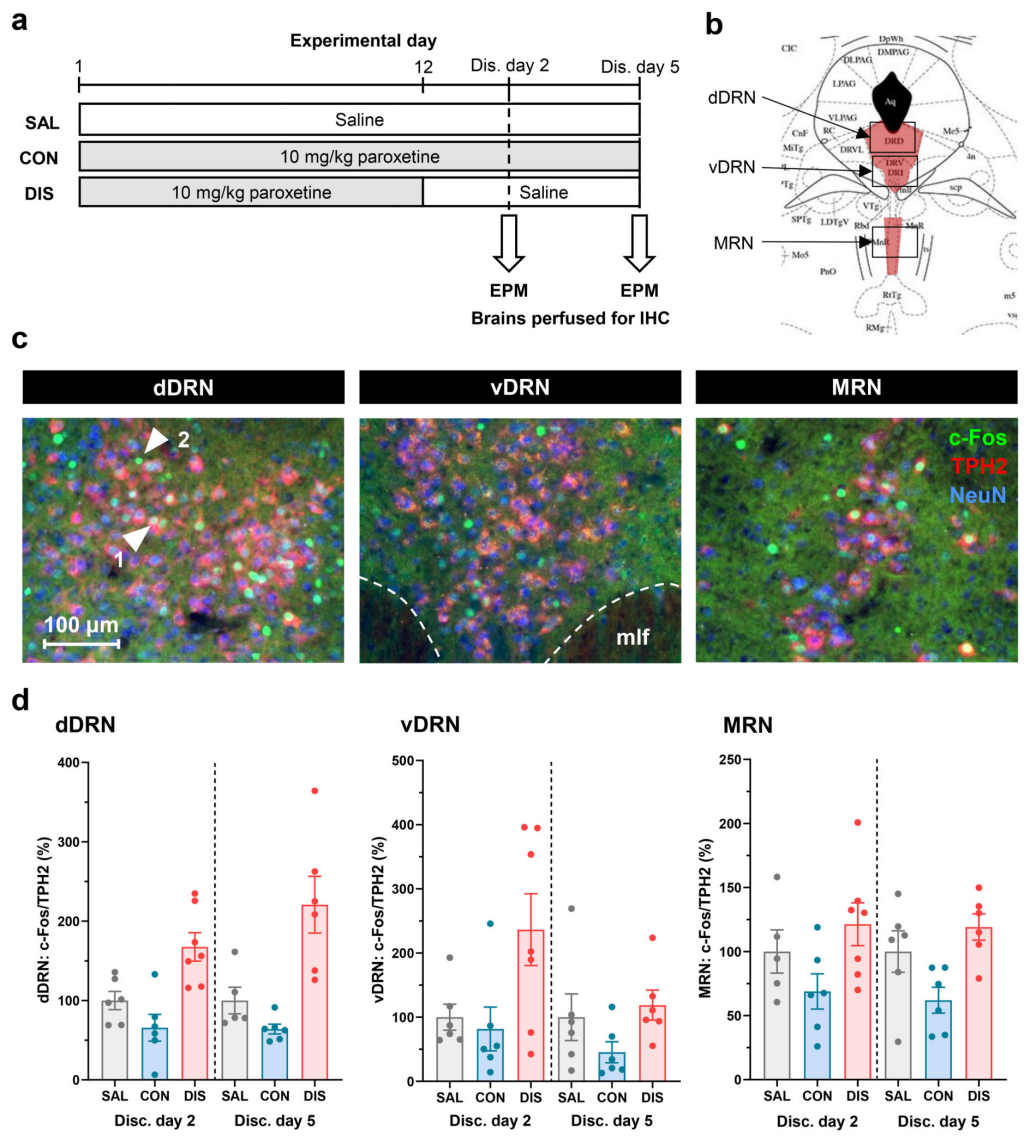
Effect of paroxetine discontinuation on c-Fos expression in 5-HT (TPH2 immunoreactive) neurons. (a) Experimental design; SAL, saline; CON, continued paroxetine; DIS, discontinuation; EPM, elevated plus maze; IHC, immunohistochemistry. (b) Illustration of cell counting areas for dorsal DRN, ventral DRN and MRN (adapted from Paxinos & Franklin (2007), AP = -4.8 mm) (c) Representative images of c-Fos, TPH2 and NeuN immunoreactivity. Arrow 1 indicates a c-Fos/TPH2 double-labelled neuron, arrow 2 indicates a c-Fos immunoreactive TPH2 immunonegative neuron. mlf, medial longitudinal fasciculus. (d) c-Fos/TPH2 double-labelled neurons in dorsal DRN (dDRN), (e) c-Fos/TPH2 double-labelled neurons in ventral DRN (vDRN), and (f) c-Fos/TPH2 double-labelled neurons in MRN on discontinuation days two (SAL n=6; CON n=6; DIS n=7) and five (SAL n=6; CON n=6; DIS n=6). Number of c-Fos/TPH2 double-labelled neurons (as a proportion of total TPH2 neurons) expressed as % of SAL group mean on each day. Mean ± SEM values are shown, with individual values indicated by dots. Data analysed with two-way ANOVA with Tukey’s post-hoc test.

Mice subjected to repeated saline, continued paroxetine and paroxetine discontinuation were tested on an EPM (5 min exposure; Fig. 1a) as described previously (Collins et al., 2022). Behavioural data from the paroxetine discontinuation experiments are presented in Collins et al. (2024) and a summary is provided in Supplementary Table 1. To determine whether EPM exposure itself increased c-Fos immunoreactivity, drug-naïve mice were exposed either to the EPM or a control environment (placement in a clean open-top cage lined with sawdust).

### 2.4 c-Fos immunohistochemistry

Ninety min after EPM exposure, mice (6-7 per group, randomly preselected within each treatment group) were deeply anaesthetised with sodium pentobarbital (100 mg/kg *i.p*.) and transcardially perfused with phosphate buffered saline (PBS), followed by a fixative solution of 4% paraformaldehyde (PFA) in PBS. Brains were stored at 4 °C in 4% PFA for 48 h, then stored in cryoprotective 30% sucrose in PBS at 4 °C. For sectioning, brains were coated in Cryo-M-Bed embedding compound and cooled to -80 °C for 45 min and then cryostat cut in the coronal plane (30 μm) at -20 °C. Free-floating sections were stored in antifreeze solution (40% PBS, 30% ethylene glycol, 30% glycerol) at -20 °C.

Three non-sequential sections were selected for immunohistochemical analysis from the following brain regions (anterior-posterior co-ordinates according to Paxinos and Franklin, 2007, Figs. 1 and 3); dorsal and ventral DRN and MRN (-4.7 mm), dorsolateral and ventrolateral PAG (-4.7 mm), PVN (-0.6 mm), BLA (-1.8 mm), dentate gyrus and CA3 regions of dorsal and ventral hippocampus (AP -1.8 mm and -3.0 mm, respectively), anterior cingulate cortex (ACC) and orbitofrontal cortex (OFC) (+2.2 mm), and prelimbic cortex (PLC) (+1.6 mm).

Sections were sequentially washed in PBS, ammonium chloride, then PBS with 0.3% TWEEN® 80 (PBS-T). Sections were blocked in PBS-T with 10% donkey serum for 1 h at room temperature prior to incubation with primary antibodies (1:1000 dilution in PBS-T with 2% donkey serum) for 1 h at room temperature then overnight at 4 °C (rabbit anti-c-Fos [EPR20769, Abcam, ab214672]; goat anti-TPH2 [Abcam, Ab121013]; mouse anti-NeuN [1B7, Abcam, Ab104224]). Sections were then washed in PBS-T before incubating with secondary antibodies (1:1000 in PBS-T with 2% donkey serum) for 2 h at room temperature (donkey anti-rabbit IgG (H+L) Alexa Flor™ 488 [Invitrogen, A21206]; donkey anti-goat IgG (H+L) Alexa Flor™ 568 [Invitrogen, A11057]; donkey anti-mouse IgG (H+L) Alexa Flor™ 647 [Invitrogen, A21202]). Sections were washed in PBS-T then PBS before mounting onto glass slides (Vectashield) and covered with a glass coverslip. Slides were stored at 4 °C before image acquisition using an epifluorescent microscopy (Olympus BX40) and ImageJ software (Micromanager v1.4) with 500 ms exposure at 10 and 20x magnification.

Following initial analyses of TPH2 immunoreactivity in the DRN, further sections containing the ventral DRN were triple-labelled for c-Fos, TPH2 and VGLUT3 immunoreactivity (primary antibodies: c-Fos and TPH2 as above; guinea pig anti-VGLUT3 [Synaptic Systems, 135 204], 1:500; secondary antibodies at 1:1000: donkey anti-rabbit as above; donkey anti-goat IgG H&L Alexa Fluor® 647 [Abcam, ab150131]; Cy™3 AffiniPure goat anti-guinea Pig IgG (H+L) Conjugate [Jackson Immune Research, 106-165-003]). In these experiments, cell nuclei were stained using DAPI at 1:1000 dilution in PBS-T with 2% donkey serum for 5 min after secondary antibody incubation.

### 2.5 c-Fos quantification

Cells were manually counted using the ImageJ Cell Counter plugin by an experimenter blind to treatment group. Brightness of the images was set and remained constant for each brain region, and contrast was not adjusted for counting. NeuN- or DAPI-positive cells in the DRN and MRN were counted for c-Fos, TPH2, c-Fos/TPH2, VGLUT3/TPH2 and c-Fos/TPH2/VGLUT3 labelling. In the other brain regions, the number of NeuN-labelled c-Fos immunoreactive cells were counted (regions of interest shown in Supplementary Figure 1). In hippocampal subregions, NeuN immunoreactivity was too dense to identify individual nuclei, so the number of c-Fos immunoreactive neurons per mm^2^ was calculated (average area of 0.22 mm^2^ and 0.19 mm^2^ per section for the dentate gyrus and CA3, respectively). Supplementary Table 2 details how the proportion of c-Fos immunoreactive neurons were calculated for each brain region, to account for variable number of neurons per region in each slice. Immunoreactivity was quantified in three sections per region per mouse, then averaged per region per mouse. Data are expressed as a percentage of the saline group mean for each discontinuation day to take into account small between-experiment variations in c-Fos immunoreactivity.

### 2.6 Statistical analysis

For discontinuation experiments, cell count data were analysed using two-way ANOVA with Tukey’s post-hoc test, with treatment and discontinuation day as between-subject factors. Cell count data for the experiment involving EPM exposure (versus cage controls) were analysed with Student’s t-test (GraphPad Prism v9). Outliers were identified and removed using ROUT analysis. All cell counting and analyses were performed by an experimenter blind to treatment/exposure group. Data are presented as mean ± SEM values, and p<0.05 is considered statistically significant.

## 3 Results

### 3.1 Paroxetine discontinuation increased c-Fos expression in DRN 5-HT neurons

Previously, we found that two but not five days after paroxetine discontinuation, male mice show evidence of increased anxiety-like behaviour on the EPM, evident as reduced time spent in and entries to the open arms, as well as reduced distance travelled on the EPM (Collins et al., 2024); see Supplementary Table 1 for summary of key results. Here, we investigated the effect of two and five days of paroxetine discontinuation on the number of c-Fos immunoreactive neurons (c-Fos immunoreactivity co-localised with nuclear NeuN) in subregions of the DRN and MRN (representative images shown in Fig. 1b). C-Fos immunoreactive neurons co-localised TPH2 in both the dorsal and ventral DRN as well as the MRN (Fig. 1c); c-Fos was also detected in DRN and MRN neurons that were immunonegative for TPH2, i.e. non-5-HT neurons (Fig. 1c).

In the dorsal DRN, paroxetine discontinuation increased the number of c-Fos immunoreactive neurons compared to both continued paroxetine and saline controls (Table 1). Specifically, paroxetine discontinuation increased the number of c-Fos/TPH2 double-labelled neurons (as a proportion of the total number of TPH2 immunoreactive neurons) compared to continued paroxetine and saline controls (main effect of treatment: F_(2,30)_=23.55 p<0.0001, post-hoc Tukey’s saline (SAL) vs discontinuation (DIS) p=0.0002, continuation (CON) vs DIS p<0.0001; Fig. 1d). This was evident as a main effect of treatment, regardless of discontinuation day (no effect of day: F_(1,30)_=1.104, p=0.3017; no treatment*day interaction: F_(2,30)_=1.270, p=0.2956; Fig. 1d).

In the ventral DRN, paroxetine discontinuation increased the overall number of c-Fos immunoreactive neurons compared to continued paroxetine and saline controls (Table 1). Paroxetine discontinuation also significantly increased the overall number of c-Fos/TPH2 double-labelled neurons compared to continued paroxetine, and there was a trend towards an increase compared to saline controls (main effect of treatment: F_(2,31)_=5.440, p=0.0094, post-hoc Tukey’s CON vs DIS p=0.0090, SAL vs DIS p=0.0894; effect of day: F_(1,31)_=3.106, p=0.0897), although there was no significant treatment*day interaction (F_(2,31)_=1.457, p=0.2485; Fig. 1d).

In contrast to the DRN, paroxetine discontinuation did not increase the number of c-Fos/TPH2 double-labelled neurons in the MRN compared to saline controls, although post-hoc tests showed that there were more in the discontinuation group than in the group receiving continued paroxetine (main effect of treatment: F_(2,30)_=7.645, p=0.0021, post-hoc Tukey’s CON vs DIS p=0.0015; no effect of day: F_(1,30)_=0.0654, p=0.7999; no interaction: F_(2,30)_=0.0287, p=0.9717; Fig. 1d). Notably, continued paroxetine reduced the number of c-Fos immunoreactive neurons compared to saline controls, and c-Fos expression returned to control levels following discontinuation (Table 1).

There were no significant effects of any treatment on the number of TPH2 expressing neurons in either the DRN or MRN (Table 1). There were generally no effects of discontinuation on the number of c-Fos positive neurons that were immunonegative for TPH2 (as a proportion of the total number of TPH2 immunonegative neurons); however, in the dorsal DRN, the number of these neurons was greater in the discontinuation versus continuation group, but this effect was not significant from saline controls (Supplementary Table 3).

Overall, two days of paroxetine discontinuation increased the number of c-Fos/TPH2 double-labelled neurons above saline-control levels in the dorsal DRN, and above both the dorsal and ventral DRN but not the MRN. This effect persisted on discontinuation day five in the dorsal DRN but not the ventral DRN.

### 3.2 Paroxetine discontinuation increased c-Fos expression in DRN 5-HT-glutamate co-releasing neurons

**Figure 2:**
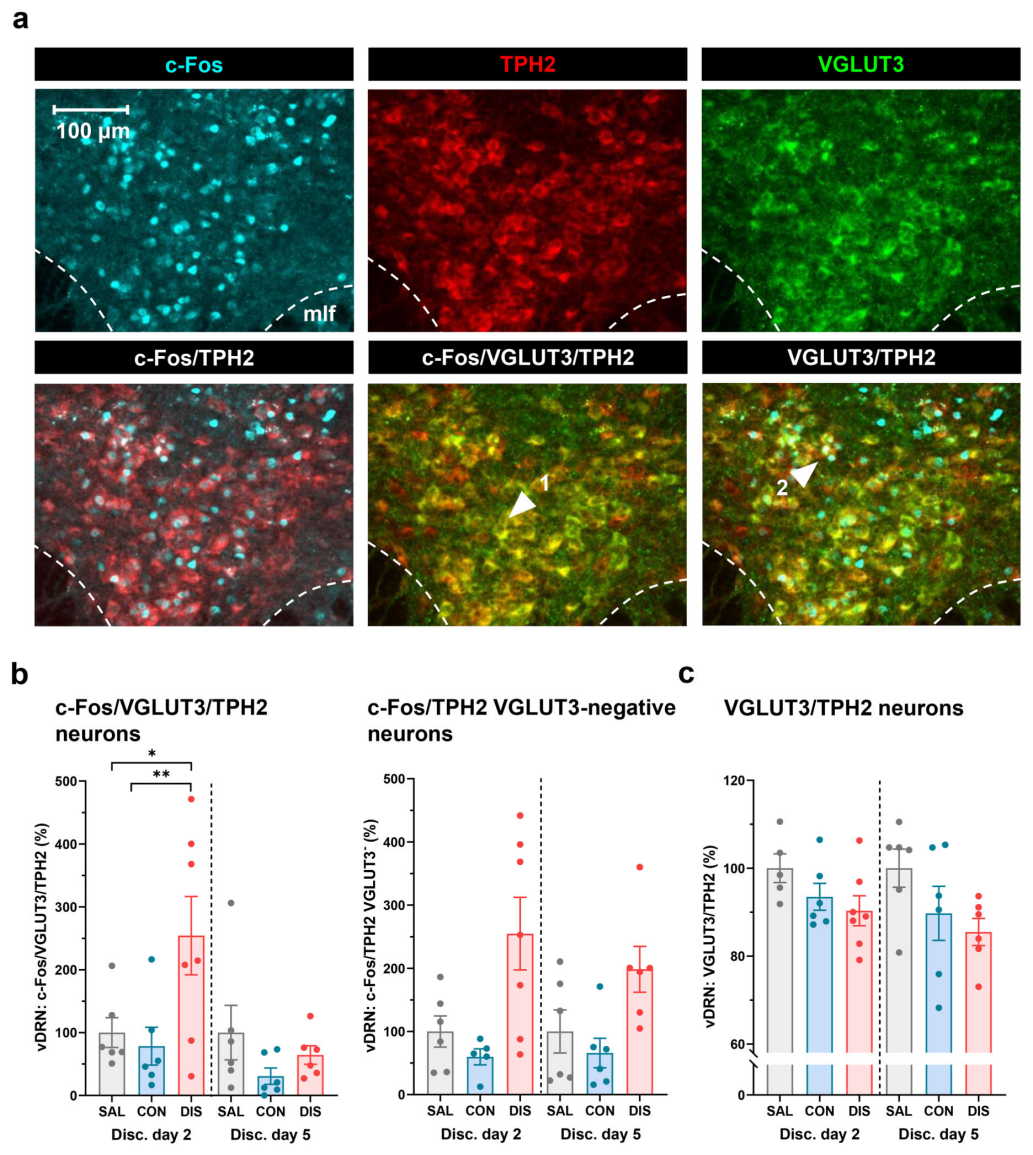
Effect of paroxetine discontinuation on c-Fos expression in 5-HT-glutamate (TPH2/VGLUT3) neurons. (a) Representative images showing c-Fos, TPH2, VGLUT3 and NeuN immunoreactive neurons in the vDRN. Arrow 1 indicates a VGLUT3/TPH2 double-labelled neuron, arrow 2 indicates a c-Fos/VGLUT3/TPH2 triple-labelled neuron. mlf, medial longitudinal fasciculus. (b) c-Fos/TPH2/VGLUT3 triple-labelled neurons and c-Fos/TPH2 VGLUT3-immunonegative neurons, (c) TPH2/VGLUT3 double-labelled neurons in ventral DRN (vDRN) on discontinuation days two (SAL n=5-6; CON n=5; DIS n=7; one significant outlier excluded from CON group) and five (SAL n=6; CON n=6; DIS n=6). Number of neurons expressed as % of SAL group mean on each day. Mean ± SEM values are shown, with individual values indicated by dots. Data analysed with two-way ANOVA with Tukey’s post-hoc test, * p<0.05, ** p<0.01.

To further investigate the phenotype of DRN neurons activated by paroxetine discontinuation, we also probed sections for VGLUT3, a marker of glutamate co-release from 5-HT neurons (Gras et al., 2002; Schäfer et al., 2002). We found the majority of neurons double-labelled for VGLUT3 and TPH2 in the ventral DRN (Fig. 2a), with sparse labelling in other DRN subregions or the MRN, in accordance with previous reports (Hioki et al., 2010; Okaty et al., 2020; Gullino et al., 2024). Importantly, immunostaining also revealed neurons triple-labelled for c-Fos, TPH2 and VGLUT3 (Fig. 2a).

Interestingly, paroxetine discontinued mice showed an increase in the number of c-Fos/VGLUT3/TPH2 triple-labelled neurons (as a proportion of the total number of VGLUT3/TPH2 neurons) (main effect of treatment: F_(2,31)_=3.883, p=0.0313, post-hoc Tukey’s CON vs DIS p=0.0245; effect of day: F_(1,31)_=6.518, p=0.0158; interaction: F_(2,31)_=3.455, p=0.0442; Fig. 2b). Post-hoc tests showed that two days of paroxetine discontinuation increased the number of c-Fos/VGLUT3/TPH2 neurons compared to both continued paroxetine and saline controls, but this effect was not evident on discontinuation day five (post-hoc Tukey’s on Fig. 2b). On the other hand, paroxetine discontinuation increased c-Fos expression in TPH2-immunopostive VGLUT3-immunonegative neurons in the ventral DRN compared to continued paroxetine and saline controls, but this effect did not differ between discontinuation days (main effect of treatment: F_(2,30)_=10.74, p=0.0003; no effect of day: F_(1,30)_=0.2996, p=0.5882; no interaction: F_(2,30)_=0.4369, p=0.6501; Fig 2b).

Although there was no effect of treatment on the number of TPH2 immunopositive neurons (see section 3.1), we found that continued paroxetine produced a modest reduction in the number of neurons co-expressing TPH2 and VGLUT3 compared to saline controls (as a proportion of the total number of TPH2 neurons), which was then significantly decreased following discontinuation (main effect of treatment: F_(2,30)_=4.509, p=0.0194, post-hoc Tukey’s SAL vs DIS p=0.0160; no effect of day: F_(1,30)_=0.7383, p=0.3970; no interaction: F_(2,30)_=0.1874, p=0.8301; Fig. 2c). Thus, two days of paroxetine discontinuation was associated with an increased number of c-Fos/VGLUT3/TPH2 triple-labelled neurons above saline control levels in ventral DRN, while the number of TPH2/VGLUT3 labelled neurons decreased across the discontinuation period.

### 3.3 Paroxetine discontinuation increased c-Fos expression in ventral hippocampus

**Figure 3:**
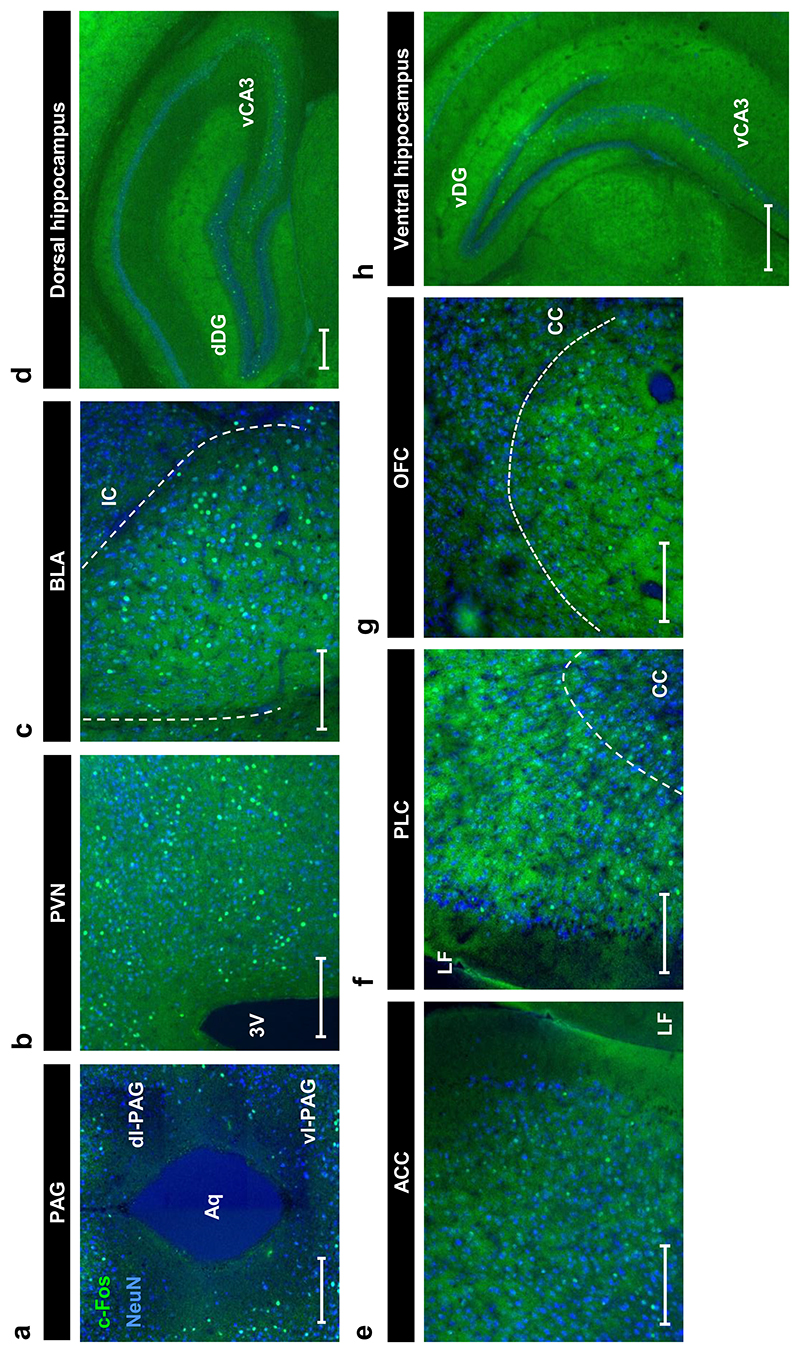
Representative images showing c-Fos immunoreactive neurons in various anxiety-related forebrain and midbrain regions. (a) Dorsolateral (dl) and ventrolateral (vl) PAG, (b) PVN, (c) BLA (d) dentate gyrus and CA3 region of dorsal hippocampus, (e) ACC, (f) PLC, (g) OFC and (h) dentate gyrus and CA3 of ventral hippocampus. C-Fos immunoreactivity (green), NeuN immunoreactivity (blue). Scale bars represent 100 μm, except for panel h where it represents 250 μm. Abbreviations as specified in main text; Aq, cerebral aqueduct; 3V, third ventricle; IC, interior capsule; LF, longitudinal fissure; CC, corpus callosum.

**Figure 4:**
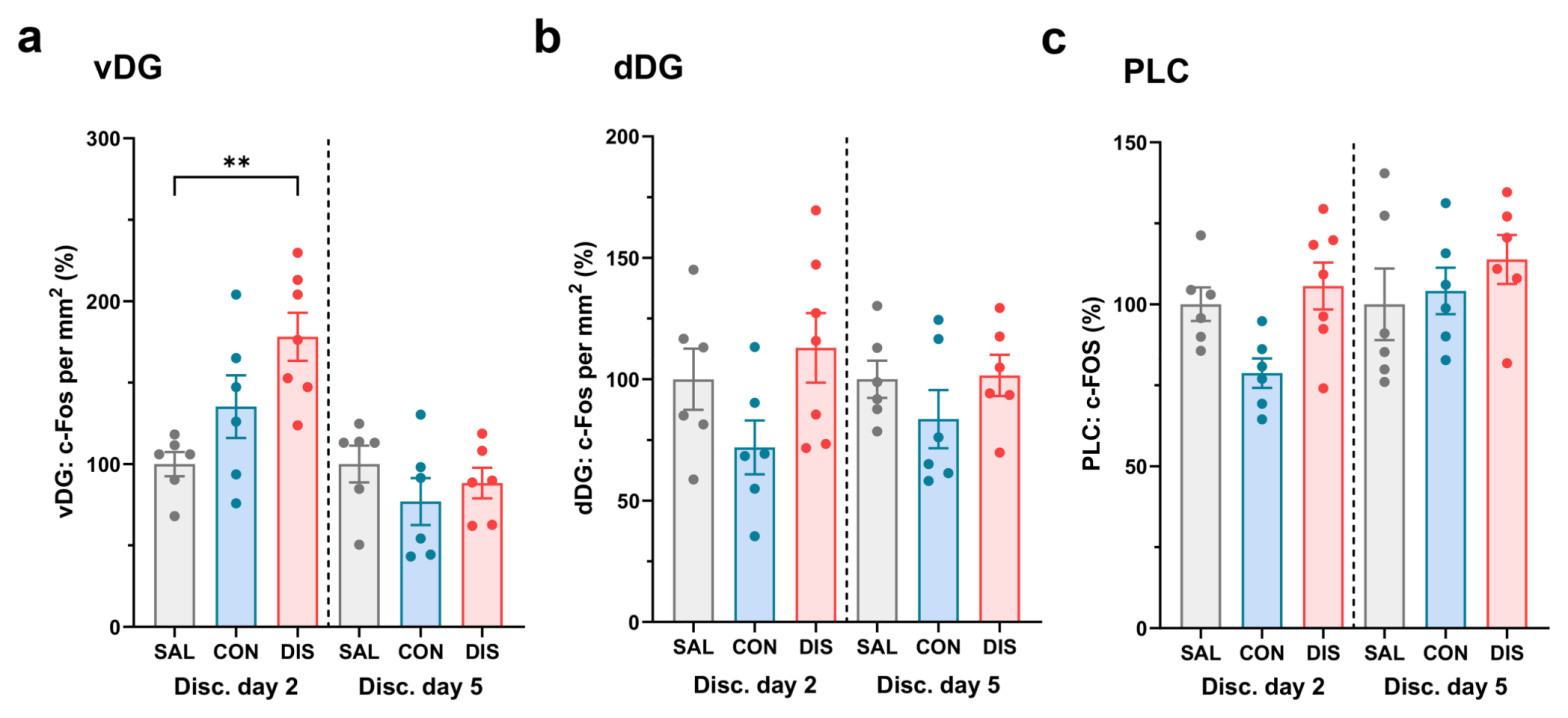
Effect of paroxetine discontinuation on c-Fos expression in hippocampus and prelimbic cortex. Number of c-Fos immunopositive neurons in (a) dentate gyrus of dorsal hippocampus (dDG), (b) dentate gyrus of ventral hippocampus (vDG) and (c) prelimbic cortex (PLC) on discontinuation days two (SAL n=6; CON n=6; DIS n=7) and five (SAL n=6; CON n=6; DIS n=6). Number of neurons expressed as % of SAL group mean on each day. Mean ± SEM values are shown, with individual values indicated by dots. Data analysed with two-way ANOVA with Tukey’s post-hoc test, ** p<0.01.

We next explored the effect of paroxetine discontinuation on c-Fos expression in the ventral hippocampus, a region associated with anxiety-related behaviour in rodents (Bannerman et al., 2003; Bannerman et al., 2004) that receives significant 5-HT innervation (e.g. McQuade and Sharp (1997); Abrams et al. (2005)). C-Fos immunoreactive neurons were detected in dentate gyrus and CA3 regions of the dorsal and ventral hippocampus (Fig. 3). Compared to saline controls, paroxetine discontinuation increased the number of c-Fos immunoreactive neurons in the dentate gyrus of the ventral hippocampus on discontinuation day two, but not on day five (main effect of treatment: F_(2,31)_=3.527, p=0.0417, post-hoc Tukey’s SAL vs DIS p=0.0477; effect of day: F_(1,31)_=20.14, p<0.0001; interaction: F_(2,31)_=5.744, p=0.0075; Fig. 4a). There was no effect of treatment on c-Fos expression in the CA3 subregion of the ventral hippocampus (Table 2). Discontinuation had no effect on the number of c-Fos-immunoreactive neurons in either the dentate gyrus (no main effect of treatment: F_(2,31)_=3.075, p=0.0605, post-hoc Tukey’s CON vs DIS p=0.0484; no effect of day: F_(1,31)_=3.390, p=0.0752; no interaction: F_(2,31)_=1.480, p=0.2433; Fig. 4b) or CA3 region (Table 2) of the dorsal hippocampus. Thus, two days of paroxetine discontinuation was associated with an increase in c-Fos immunoreactive neurons in the ventral but not dorsal hippocampus.

### 3.4 Paroxetine discontinuation did not increase c-Fos expression in other anxiety-related brain regions

We then explored c-Fos expression in other anxiety-related brain regions, starting with ACC, PLC, OFC (Fig. 3e-g), and the BLA (Fig. 3c). In the PLC, continued paroxetine reduced c-Fos expression compared to saline controls, but paroxetine discontinuation did not significantly differ from continued paroxetine or saline controls (main effect of treatment: F_(2,31)_=3.554, p=0.0408, post-hoc Tukey’s CON vs DIS p=0.0445; no effect of day: F_(1,31)_=0.0002, p=0.9899; no interaction: F_(2,31)_=0.5051, p=0.6083; Fig. 4c). There were no effects of paroxetine discontinuation on the number of c-Fos immunoreactive neurons in the ACC, OFC or BLA (Table 2).

The effect of paroxetine discontinuation on c-Fos immunoreactive neurons was also quantified in the PAG and PVN, two regions associated with stress and anxiety-like behaviours (Deakin and Graeff, 1991; Graeff et al., 1993; Jiang et al., 2019). Although there was a main effect of treatment on the number of c-Fos immunoreactive neurons in the ventrolateral PAG, post-hoc tests found no significant differences between groups (Table 2). There were also no effects of paroxetine discontinuation on c-Fos in the dorsolateral PAG or PVN (Table 2). Taken together, the above findings suggest that two days of paroxetine discontinuation did not alter the number of c-Fos immunoreactive neurons in ACC, PLC, OFC, BLA, PVN or PAG.

### 3.5 Effect of EPM exposure alone on c-Fos expression

A potential explanation for the limited number of brain regions exhibiting a c-Fos response to paroxetine discontinuation is that EPM exposure itself increased c-Fos expression in certain anxiety-related regions, such that discontinuation could not increase c-Fos expression further (i.e. ceiling effect, see Discussion). To assess this possibility, a separate cohort of drug-naïve mice was exposed to either the EPM or a clean homecage for 5 min before tissue collection.

EPM exposure (compared to homecage exposure) did not alter the number of c-Fos/TPH2 double-labelled neurons in either the dorsal DRN (t_(12)_=0.9577, p=0.3571) or ventral DRN (t_(9)_=0.0894, p=0.9308; Suppl. Fig. 2a-b), or the MRN (t_(12)_=1.245, p=0.2368; Suppl. Fig. 2c). In contrast, EPM exposure increased the number of c-Fos immunoreactive neurons in the dentate gyrus of both the dorsal (t_(12)_=2.448, p=0.0148; Suppl. Fig. 2d) and ventral hippocampus (t_(12)_=2.295, p=0.0405; Suppl. Fig. 2e), but not in the PLC (t_(12)_=0.1971, p=0.8471; Suppl. Fig. 2f) or BLA (t_(12)_=0.1942, p=0.8492; Suppl. Fig. 2g).

## 4 Discussion

Abrupt cessation of SSRI therapy can produce a debilitating discontinuation syndrome, which includes an increase in anxiety that has also been detected in studies of SSRI discontinuation in rodents (see Introduction). For example, we recently found that mice discontinued from paroxetine or citalopram exhibited a transient increase in anxiety-like behaviour, i.e. present at two but not five days post-discontinuation (Collins et al., 2024). Here, we used c-Fos immunohistochemistry to test whether paroxetine discontinuation would, as predicted, activate anxiety-related midbrain and forebrain circuitry. Indeed, we found that paroxetine discontinuation increased the number of TPH2-expressing (5-HT) neurons immunoreactive for c-Fos in the various subregions of the DRN, indicating 5-HT neuron activation, an effect often associated with increased anxiety. Paroxetine discontinuation also increased the number of c-Fos immunoreactive neurons in ventral hippocampus, another anxiety-related brain region. Both effects were detected two days post-discontinuation, with the DRN showing increased c-Fos expression at five days. Surprisingly, however, paroxetine discontinuation was not associated with increased c-Fos expression in other anxiety- or stress-related brain regions including the PFC, BLA, PVN and PAG. These findings suggest that, under the present conditions, paroxetine discontinuation causes a short-lasting activation of some, albeit limited, components of the anxiety circuitry.

We found that paroxetine discontinuation increased the number of c-Fos/TPH2 double-labelled neurons in both the dorsal and ventral DRN, indicating excitation of the main source of 5-HT innervation to the forebrain. This effect was specific to 5-HT neurons as there was limited evidence of increased c-Fos expression in TPH2 immunonegative (non-5-HT) neurons. This finding is consistent with previous reports that cessation of SSRI administration resulted in a rebound increase 5-HT synthesis, metabolism and release in forebrain regions including hippocampus (Caccia et al., 1993; Trouvin et al., 1993; Stenfors and Ross, 2002; Bosker et al., 2010; Guiard et al., 2012; Collins et al., 2024). The latter effect of discontinuation may arise from the sudden relief of 5-HT autoreceptor inhibition that is likely present under constant exposure to an SSRI (Sharp and Collins, 2024). In contrast to the DRN, paroxetine discontinuation did not increase c-Fos expression in 5-HT neurons in the MRN, indicating that not all 5-HT neuron subpopulations were activated.

Evidence is accumulating for 5-HT neuron heterogeneity at multiple levels, ranging from developmental origin and neuroanatomical connectivity to molecular phenotype and physiological function (Okaty et al., 2019). For example, DRN subregions as well as the MRN differ in their neuronal inputs, and this may contribute to the differential activation of 5-HT neuron subpopulations. Also, 5-HT neurons differ in levels of 5-HT transporter and autoreceptor expression (Huang et al., 2019), which may make some 5-HT neurons more sensitive to the effects of paroxetine discontinuation than others.

Interestingly, further immunohistochemical analysis revealed that in the ventral DRN discontinuation increased c-Fos in neurons double-labelled for TPH2 and VGLUT3 on discontinuation day two; whereas, non-VGLUT3-expressing TPH2 neurons were broadly activated throughout the discontinuation period, similar to the effects seen in the dorsal DRN (which has low levels of 5-HT-gluatamte co-expression). This finding implies activation of a subpopulation of 5-HT-glutamate co-releasing neurons on discontinuation day two that had subside by day five. In addition, a chance finding was that the number of VGLUT3 TPH2 double-labelled neurons decreased during paroxetine discontinuation. Given the number of TPH2 immunopositive neurons did not change, this suggests that VGLUT3 expression was downregulated following paroxetine discontinuation, in which case the balance of 5-HT and glutamate co-release would shift towards 5-HT. Such a scenario would be in keeping with recent evidence that 5-HT neurons have the capacity to switch towards a glutamatergic phenotype (Boulland et al., 2004; Gras et al., 2005; Dulcis et al., 2013; Spitzer, 2015; Li et al., 2020; Prakash et al., 2020), and speculation that SSRIs may change the balance of 5-HT and glutamate co-transmission (Fischer et al., 2015; Gullino et al., 2024).

Several lines of evidence suggest there may be a causal link between discontinuation-induced activation of DRN 5-HT neurons and increased anxiety. Firstly, the same two day paroxetine discontinuation protocol that here increased the number of c-Fos/TPH2 doubled-labelled neurons was previously shown to increase anxiety-like behaviour on the EPM (Collins et al., 2022; Collins et al., 2024). Secondly, previous studies have shown that administration of anxiogenic drugs evoked c-Fos expression in the DRN (Singewald and Sharp, 2000). Thirdly, *in vivo* electrophysiological studies have shown that exposure of mice to an anxiogenic stimulus increases the firing of DRN 5-HT neurons (e.g. Cohen et al. (2015); Ren et al. (2018)). Finally, and importantly, optogenetic and chemogenetic activation of DRN 5-HT neurons and their forebrain projections was found to provoke a 5-HT receptor-mediated increase in anxiety-like behaviour, including on the EPM (Vicente et al., 2008; Marcinkiewcz et al., 2016; Ren et al., 2018); although see (Ohmura et al., 2014), which suggests that optogenetic activation of the MRN, but not DRN, increases anxiety on the EPM in mice. Moreover, it is important to note that while five days of paroxetine discontinuation increased the number of c-Fos/TPH2 double-labelled neurons in the DRN, the same treatment did not evoke anxiety-like behaviour on the EPM (Collins et al 2024). Thus, discontinuation-induced activation of DRN 5-HT neurons may not itself be sufficient to evoke anxiety.

It is also plausible that increased activity of 5-HT-glutamate co-releasing neurons contributes to the behavioural effects of discontinuation. As with paroxetine discontinuation, we recently observed that acute swim stress increased the expression of c-Fos in DRN neurons that co-localised VGLUT3 and TPH2 (Gullino et al., 2024). Altered 5-HT-glutamate co-release has also been implicated in the behavioural response to chronic stress (Prakash et al., 2020), as well as during acquisition of generalised fear following acute stress (Li et al., 2024). Moreover, chemogenetic activation of the projection from the ventral DRN 5-HT to the OFC, which is rich in 5-HT-glutamate co-releasing neurons, facilitated stress coping behaviour (Ren et al., 2018). It is therefore possible that on the one hand paroxetine discontinuation activates anxiety-provoking 5-HT neurons in the dorsal DRN whilst also activating 5-HT-glutamate neurons in the ventral DRN to reduce the effects of an aversive experience, which was critically only evident on day two.

Our observation of increased c-Fos expression in DRN 5-HT neurons was accompanied by increased c-Fos expression in the DG of the ventral hippocampus two days after paroxetine discontinuation. Activation of the ventral hippocampus may well contribute to an anxiety-provoking effect of paroxetine discontinuation because there is a well-established link between this region and anxiety (Bannerman et al., 2004), with its activity promoting behavioural inhibition (McNaughton and Gray, 2000). As with the DRN, anxiety-provoking situations are reported to increase c-Fos expression in the ventral hippocampus (Hale et al., 2008; Peng et al., 2019; Vo et al., 2021). Moreover, pharmacological activation of the ventral hippocampus increased anxiety-like behaviour on the EPM (Ohmura et al., 2020; Vo et al., 2021). Finally, here, c-Fos expression in the ventral hippocampus increased on discontinuation day two but not five which follows the timing of anxiety-like behaviour (see above), providing a further link to the anxiety state.

Although it generally considered that the major 5-HT projection to the ventral hippocampus arises from the MRN, there is also 5-HT innervation from the DRN (Azmitia and Segal, 1978; Sharp et al., 1990; McQuade and Sharp, 1997; Abrams et al., 2005). It is therefore possible that the hippocampal c-Fos response to discontinuation is a consequence of DRN 5-HT neuron activation. This pathway has also been linked to behavioural inhibition in mice (McNaughton and Gray, 2000; Teissier et al., 2015; Correia et al., 2017; Ren et al., 2018), and optogenetic and chemogenetic activation of the DRN induces changes in ventral hippocampal activity in awake mice (Giorgi et al., 2017; Hamada et al., 2024).

Surprisingly, aside from the DRN and ventral hippocampus, other brain regions that we predicted might be activated by an anxiety-like state did not exhibit increased c-Fos expression in response to paroxetine discontinuation. This included the MRN, which has been associated with anxiety mechanisms; indeed, optogenetic studies typically show that MRN, rather than DRN, activation drives anxiety-like behaviour on the EPM (Ohmura et al., 2014; Ohmura et al., 2020). Moreover, there was no evidence of activation above saline control levels of PFC subregions, BLA, PVN, or PAG – these are all anxiety-linked regions that typically show increased c-Fos immunoreactivity in response to exposure to anxiogenic drugs and other anxiety-provoking stimuli (Duncan et al., 1996; Singewald and Sharp, 2000; Linden et al., 2003; Singewald et al., 2003; Salomé et al., 2004; Ait Bali et al., 2022).

One explanation for the limited number of brain regions showing a c-Fos response to paroxetine discontinuation could be that prior to brain tissue extraction we exposed mice to the EPM. This experience by itself may have increased c-Fos expression such that further increases would be undetectable. However, this is unlikely since separate experiments on treatment-naïve mice found that EPM exposure (under the present conditions) only increased c-Fos expression in the hippocampus. Rather, a more plausible explanation is that the effects of paroxetine discontinuation on anxiety circuitry were limited in magnitude and/or nature due to prior placement on the EPM. Thus, unaltered c-Fos expression in BLA may reflect this region’s roles in active avoidance (e.g. directed escape responses) and conditioned or learned anxiety (Graeff et al., 1993; McNaughton and Gray, 2000; McHugh et al., 2004), which are not assessed on the EPM, which assessed unconditioned anxiety. Similarly, the lack of change in c-Fos expression in PFC might reflect that EPM performance does not engage mechanisms of behavioural control based on prior knowledge and experiences that this region provides (Jacobs and Moghaddam, 2021). It is possible that paroxetine discontinuation combined with exposure to other anxiety-provoking contexts/environments might reveal activation of c-Fos in other regions. Additionally, despite evidence that paroxetine discontinuation activated the ascending 5-HT pathways, this effect by itself might not be expected to increase c-Fos expression in forebrain regions; recent rodent neuroimaging data suggest that 5-HT neuron activation can both increase and decrease activity in cortical and other forebrain regions, highlighting the neuromodulatory role of 5-HT (Grandjean et al., 2019; Hamada et al., 2024). Since the PAG and PVN play key roles in panic and stress mechanisms (Singewald and Sharp, 2000; Ferguson et al., 2008), the lack of changes in c-Fos immunoreactivity in the regions suggests that paroxetine discontinuation is not inducing a general stress- or panic-like state. Nonetheless, a greater sample size of this study may have increased our ability to detect subtle changes in c-Fos expression in less responsive regions.

It may also be valuable to examine c-Fos expression at intermediate and later timepoints following paroxetine discontinuation. We previously observed anxiety-like behaviour on day two, which subsided by day five, but the trajectory of behavioural and c-Fos changes between these points remains unclear. Additionally, altered sleep structure persisted up to seven days post-discontinuation (Collins et al., 2023), suggesting that c-Fos expression may also remain altered until this time. Future studies should investigate the time course of c-Fos expression throughout the first week after discontinuation to better understand how increased regional activity changes and resolves.

Finally, there are interesting parallels between the present findings and reported changes in c-Fos expression following withdrawal from other psychotropic drugs. For example, morphine and amphetamine withdrawal in rodents both increased c-Fos expression in DRN and hippocampus (Hayward et al., 1990; Bhat et al., 1992; Stornetta et al., 1993; Beckmann et al., 1995; Georges et al., 2000; Tanaka et al., 2017), and heightened anxiety-like behaviour (Vuong et al., 2010; Ozdemir et al., 2024). Although caution must be taken drawing parallels between SSRI discontinuation and the withdrawal states of other psychotropic drugs, they may activate similar anxiety circuitry.

In conclusion, the current findings suggest that two days of paroxetine discontinuation activated 5-HT neurons in the DRN, including 5-HT-glutamate co-expressing neurons, as well as neurons in ventral hippocampus. These findings support the hypothesis developed from previous behavioural and neurochemical studies that the behavioural effects of SSRI discontinuation are associated with activation of specific anxiety-promoting circuitry. Although under the present conditions the effects seen here were limited to DRN 5-HT neurons and hippocampus, this circuitry may contribute to symptoms such as increased anxiety that form part of the SSRI discontinuation syndrome in patients.

## Supplementary Material

Supplementary

## Figures and Tables

**Table 1:** Effect of paroxetine discontinuation on c-Fos expression in the dorsal DRN (dDRN), ventral DRN (vDRN) and MRN. Data show the number of c-Fos or TPH2 immunoreactive neurons in saline (SAL), continued paroxetine (CON) and paroxetine discontinuation (DIS) groups, expressed as a % of the saline control group for each discontinuation day. Mean ± SEM values, n=6-7/group. Data analysed using two-way ANOVA with post-hoc Tukey’s test, bold values represent statistically significant effects. Post-hoc Tukey’s for significant effects of treatment: * SAL vs DIS p=0.0007, CON vs DIS p<0.0001; † SAL vs DIS p=0.0382, CON vs DIS p=0.0017; †† SAL vs CON p=0.0458, CON vs DIS p=0.0126.

Brain region	Discontinuation day 2			Discontinuation day 5			Effect of treatment	Effect of day	Treatment*day interaction
	SAL (%)	CON (%)	DIS (%)	SAL (%)	CON (%)	DIS (%)			
dDRN									
c-Fos	100.0 ± 5.8	63.7 ± 12.9	131.4 ± 12.9	100.0 ± 7.6	92.5 ± 2.5	152.7 ± 10.87	**F_(2,30)_=22.13** p<0.0001 *	F_(1,30)_=4.168, p=0.0501	F_(2,30)_=1.057, p=0.3600
TPH2	100.0 ± 10.6	102.4 ± 9.5	102.3 ± 5.6	100.0 ± 4.6	91.7 ± 9.6	110.3 ± 5.6	F_(2,30)_=0.5851, p=0.5635	F_(1,30)_=0.016 3, p=0.8991	F_(2,30)_=0.5819, p=0.5652
vDRN									
c-Fos	100.0 ± 18.7	87.7 ± 20.6	165.6 ± 33.2	100.0 ± 26.3	71.40 ± 15.8	113.3 ± 8.9	**F_(2,31)_=3.672,** **p=0.0370 †**	F_(1,31)_=1.520, p=0.0.2270	F_(2,31)_=0.7084, p=0.5002
TPH2	100.0 ± 9.0	96.6 ± 6.1	112.0 ± 8.1	100.0 ± 6.1	89.6 ± 2.7	113.5 ± 6.4	F_(2,31)_=3.177, p=0.0556	F_(1,31)_=0.079 4, p=0.7799	F_(2,31)_=0.1615, p=0.8515
MRN									
c-Fos	100.0 ± 7.3	65.4 ± 6.9	104.2 ± 10.1	100.0 ± 11.4	81.5 ± 7.3	95.6 ± 10.6	**F_(2,30)_=5.383,** **p=0.0101 ††**	F_(1,30)_=0.107 5, p=0.7452	F_(2,30)_=0.9301, p=0.4056
TPH2	100.0 ± 4.4	95.9 ± 12.9	95.9 ± 9.8	100.0 ± 13.0	97.0 ± 8.7	109.3 ± 7.5	F_(2,30)_=0.1646, p=0.8490	F_(1,30)_=0.287 6, p=0.5957	F_(2,30)_=0.2348, p=0.7922

**Table 2:** Effect of paroxetine discontinuation on c-Fos expression in anxiety-related forebrain and midbrain regions. Data show the number of c-Fos immunoreactive neurons in saline (SAL), continued paroxetine (CON) and paroxetine discontinuation (DIS) groups expressed as a % of saline controls. Mean ± SEM values, n=6-7/group. Abbreviations: d, dorsal; v, ventral; PFC, prefrontal cortex; ACC, anterior cingulate cortex; OFC, orbitofrontal cortex; BLA, basolateral amygdala; PAG, periaqueductal grey; PVN, paraventricular nucleus. Data analysed using two-way ANOVA with post-hoc Tukey’s test, bold values represent statistically significant effects. * ANOVA showed a significant effect of treatment but there were no significant post-hoc comparisons.

Brain region	Discontinuation day 2			Discontinuation day 5			Effect of treatment	Effect of day	Treatment*day interaction
	SAL (%)	CON (%)	DIS (%)	SAL (%)	CON (%)	DIS (%)			
Hippocampus									
dCA3	100.0 ± 3.9	70.1 ± 6.8	104.4 ± 11.9	100.0 ± 14.1	106.1 ± 11.0	103.7 ± 5.8	F_(2,31)_=1.116, p=0.3405	F_(1,31)_=1.683, p=0.2041	F_(2,31)_=1.776, p=0.1861
vCA3	100.0 ± 10.9	77.7 ± 8.7	96.2 ± 7.5	100.0 ± 10.9	89.4 ± 10.8	111.5 ± 12.77	F_(2,31)_=1.847, p=0.1746	F_(1,31)_=0.971 9, p=0.3318	F_(2,31)_=0.2558, p=0.7759
PFC									
ACC	100.0 ± 18.4	52.9 ± 3.8	115.9 ± 17.5	100.0 ± 20.5	93.6 ± 18.1	102.7 ± 22.6	F_(2,31)_=1.835, p=0.1765	F_(1,31)_=0.330 3, p=0.5696	F_(2,31)_=1.036, p=0.3668
OFC	100.0 ± 9.9	69.2 ± 13.0	79.5 ± 11.4	100.0 ± 21.6	69.7 ± 11.2	89.1 ± 13.1	F_(2,31)_=2.035, p=00.1478	F_(1,31)_=0.076 4, 0.7841	F_(2,31)_=0.0658, p=0.9365
BLA	100.0 ± 20.2	94.1 ± 10.2	132.5 ± 16.3	100.0 ± 7.8	106.9 ± 26.8	108.6 ± 10.6	F_(2,31)_=0.8491, p=0.4375	F_(1,31)_=0.062 8, p=0.8039	F_(2,31)_=0.5326, p=0.5923
PAG									
Dorsolateral PAG	100.0 ± 8.2	65.5 ± 11.9	94.1 ± 14.2	100.0 ± 6.2	100.7 ± 13.3	93.4 ± 5.9	F_(2,31)_=1.065, p=0.33570	F_(1,31)_=1.480, p=0.2329	F_(2,31)_=1.561, p=0.2259
Ventrolateral PAG	100.0 ± 6.8	96.4 ± 11.4	123.0 ± 11.5	100.0 ± 8.1	96.1 ± 7.0	118.3 ± 9.6	**F_(2,31)_=3.477,** p=0.0434 *	F_(1,31)_=0.040 6, p=0.8415	F_(2,31)_=0.0338, p=0.9668
PVN	100.0 ± 3.7	102.3 ± 10.1	122.3 ± 12.7	100.0 ± 6.8	115.8 ± 21.7	104.5 ± 8.1	F_(2,31)_=0.5545, p=0.5799	F_(1,31)_=0.018 1, p=0.8938	F_(2,31)_=0.7456, p=0.4828

## Data Availability

Data will be uploaded to a repository upon acceptance of the manuscript for publication and will be linked in the text.

## Abbreviations

5-HT: 5-hydroxytryptamine (serotonin)
5-HIAA: 5-hydroxyindoleacetic acid
ACC: Anterior cingulate cortex
BLA: Basolateral amygdala
DRN: Dorsal raphe nucleus
EPM: Elevated plus maze
MRN: Median raphe nucleus
OFC: Orbitofrontal cortex
PAG: Periaqueductal grey
PFC: Prefrontal cortex
PLC: Prelimbic cortex
PVN: Paraventricular nucleus of the hypothalamus
SSRI: Selective serotonin reuptake inhibitor
TPH2: Tryptophan hydroxylase 2
VGLUT3: Vesicular glutamate transporter 3
