# Supplementary material for: Increased c-Fos immunoreactivity in anxiety-related brain regions following paroxetine discontinuation
Source: Neuropharmacology. Author manuscript; Available in PMC 2026 Jan 29. (PMC7618679; doi:10.1016/j.neuropharm.2025.110541)
Supplement: Supplementary [file EMS212194-supplement-Supplementary.docx]

## Supplementary Information

**Supplementary Table 1 Effect of paroxetine discontinuation on anxiety-like behaviour on the EPM in male mice.** Key readouts of behaviour on the EPM in saline (SAL), continued paroxetine (CON) and paroxetine discontinuation (DIS) groups when tested two and five days after paroxetine discontinuation. Mean ± SEM values, n=12/group. Data analysed with one-way ANOVA, bold values represent statistically significant effects. Post-hoc Fisher’s Least Significant Difference, * SAL vs DIS p<0.01; † CON vs DIS p<0.01. Data published in graphical form in Collins et al. (2024).

| EPM parameter | SAL | CON | DIS | One-way ANOVA |
| --- | --- | --- | --- | --- |
| Discontinuation day 2 |  |  |  |  |
| Time in open arms (s) | 31.6 ± 4.1 | 37.9 ± 5.4 | 12.9 ± 2.1  * † | **F_(2,33)_ = 9.902, p = 0.0004** |
| Number of entries to open arms (s) | 5.7 ± 0.7 | 6.6 ± 0.7 | 3.3 ± 0.4  * † | **F_(2,33)_  = 7.708, p = 0.0018** |
| Total distance travelled (cm) | 12.0 ± 0.5 | 12.3 ± 0.6 | 9.7 ± 0.4  * † | **F_(2,33)_  = 7.415, p = 0.0022** |
| Discontinuation day 5 |  |  |  |  |
| Time in open arms (s) | 30.7 ± 7.1 | 17.1 ± 5.1 | 28.2 ± 5.6 | F_(2,33)_  = 1.451, p = 0.2490 |
| Number of entries to open arms (s) | 4.1 ± 0.6 | 2.9 ± 0.6 | 4.3 ± 0.8 | F_(2,33)_  = 1.100, p = 0.3449 |
| Total distance travelled (cm) | 9.2 ± 0.6 | 8.3 ± 0.9 | 10.7 ± 0.8 | F_(2,33)_  = 2.588, p = 0.0908 |


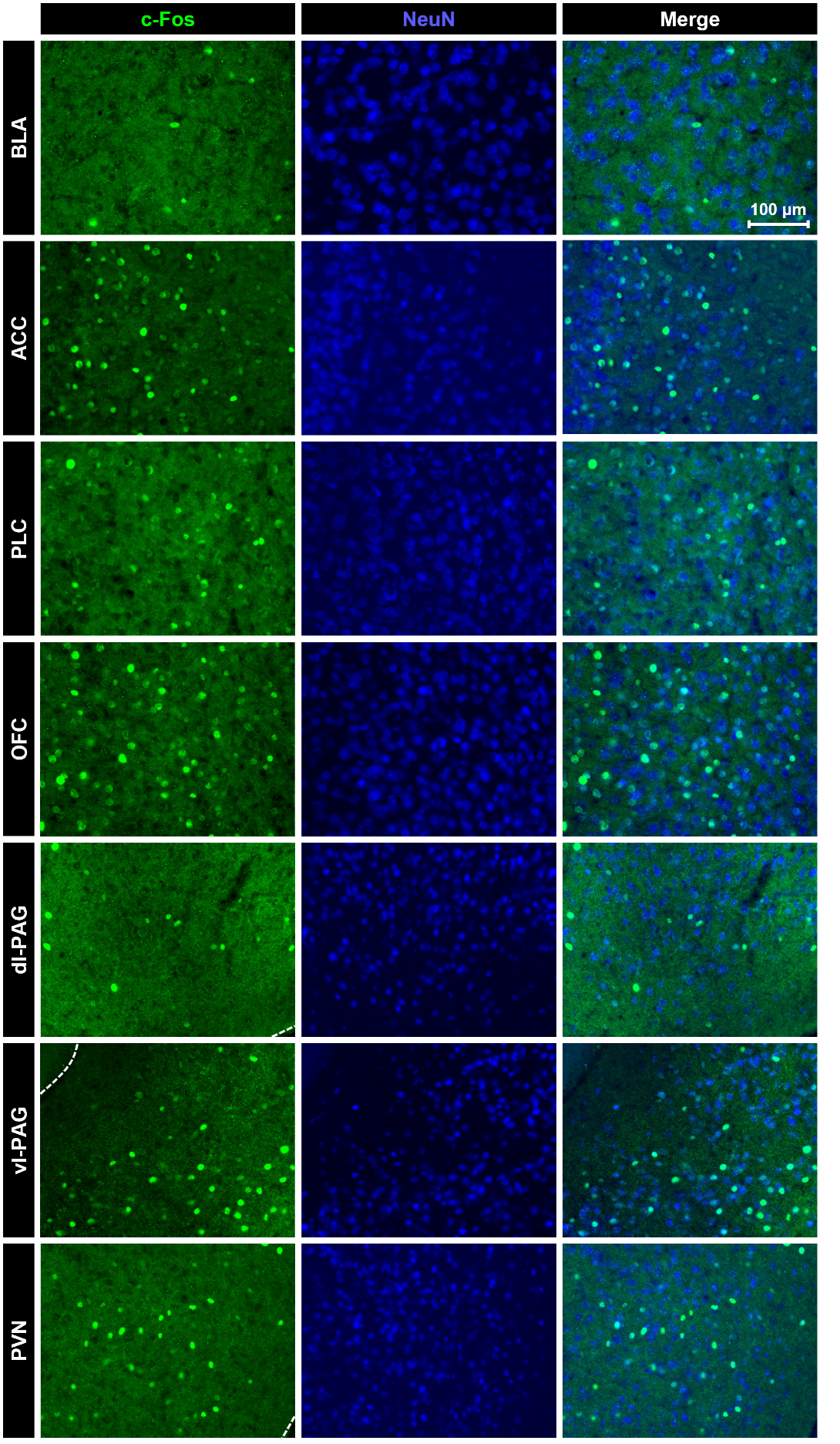


**Supplementary Figure 1 Example images and cell counting details.** Example images of the BLA, ACC, PLC, OFC, dl-PAG, vl-PAG and PVN that were used for cell counting. BLA – 10x magnification images were taken as in *Fig. 3c*, imaged for counting at 20x magnification at same location, cells counted in whole image. ACC – 10x magnification images were taken as in *Fig. 3f*, imaged for counting at 20x magnification at same location, cells counted in whole image. PLC – 10x magnification images were taken as in *Fig. 3f*, imaged for counting at 20x magnification at same location or moving medially to avoid the corpus callosum in the image, cells counted in whole image. OFC – 10x magnification images were taken as in *Fig. 3g*, imaged for counting at 20x magnification below the anterior corpus callosum, cells counted in whole image. dl-PAG – 20x magnification images taken with the aqueduct just visible in the bottom left or bottom right of the image (dotted line indicates edge of aqueduct), location as shown in *Fig. 2a*, cells counted in whole image. vl-PAG – 20x magnification images taken with the aqueduct just visible in the top left or top right of the image (dotted line indicates edge of aqueduct), location as shown in *Fig. 2a*, cells counted in whole image. PVN – 10x magnification images were taken as in *Fig. 3b*, 20x magnification images taken with third ventricle just visible to the left or right of the image (dotted line indicates edge of third ventricle), cells counted in whole image.

**Supplementary Table 2 Quantification of cell counting.** Cell counts of neurons shown in left-hand column were calculated as % of neurons demonstrating immunoreactivity for proteins shown in middle column. Relevant figure/table shown in right-hand column.

| **Percentage (%) of neurons** | **Calculation** | **Figure/Table reference** |
| --- | --- | --- |
| c-Fos | c-Fos / NeuN | Table 1, Table 2, Fig. 4 |
| c-Fos/TPH2 | (c-Fos TPH2) / (TPH2) | Fig. 1 |
| c-Fos/TPH2 | (c-Fos TPH2) / (TPH2) | Suppl. Table 2 |
| TPH2 | TPH2 / NeuN | Table 1 |
| c-Fos/VGLUT3/TPH2 | (c-Fos VGLUT3 TPH2) / (VGLUT3 TPH2 DAPI) | Fig. 2 |
| vGLUT3/TPH2 | (VGLUT3 TPH2) / (TPH2 DAPI) | Fig. 2 |

**Supplementary Table 3 Effect of paroxetine discontinuation on number of c-Fos immunoreactive TPH2 immunonegative neurons in the dorsal DRN, ventral DRN and MRN.** Number of neurons on discontinuation days two (SAL n=5-6; CON n=6; DIS n=7) and five (SAL n=6; CON n=6; DIS n=6). Number of neurons expressed as % of SAL group mean on each day. Mean ± SEM values. Data analysed with two-way ANOVA with Tukey’s post-hoc test. Significant effect of treatment, post-hoc ** CON vs DIS p=0.0192.

| **Region** | **Discontinuation day 2** | | | **Discontinuation day 5** | | | **Effect of treatment** | **Effect of day** | **Treatment* day interaction** |
| --- | --- | --- | --- | --- | --- | --- | --- | --- | --- |
|  | **SAL (%)** | **CON (%)** | **DIS (%)** | **SAL (%)** | **CON (%)** | **DIS (%)** |  |  |  |
| dDRN | 100.0 ± 11.5 | 57.3 ± 7.9 | 89.0 ± 10.0 | 100.0 ± 6.2 | 104.0 ± 7.1 | 143.7 ± 19.5 | **F_(2,30)_= 4.183, p=0.0253 **** | F_(1,30)_= 10.86, p=0.0026 | F_(2,30)_= 2.684, p=0.0852 |
| vDRN | 100.0 ± 16.7 | 87.8 ± 18.0 | 121.9 ± 13.3 | 100.0 ± 17.4 | 83.7 ± 16.9 | 110.3 ± 5.6 | F_(2,31)_= 1.318, p=0.2822 | F_(1,31)_= 0.1139, p=0.7338 | F_(2,31)_= 0.0491, p=0.9522 |
| MRN | 100.0 ± 14.3 | 63.0 ± 9.4 | 101.3 ± 10.5 | 100.0 ± 9.8 | 89.1 ± 6.5 | 81.2 ± 10.4 | F_(2,30)_= 2.243, p=0.1237 | F_(1,30)_= 0.0482, p=0.8277 | F_(2,30)_= 2.207, p=0.1276 |


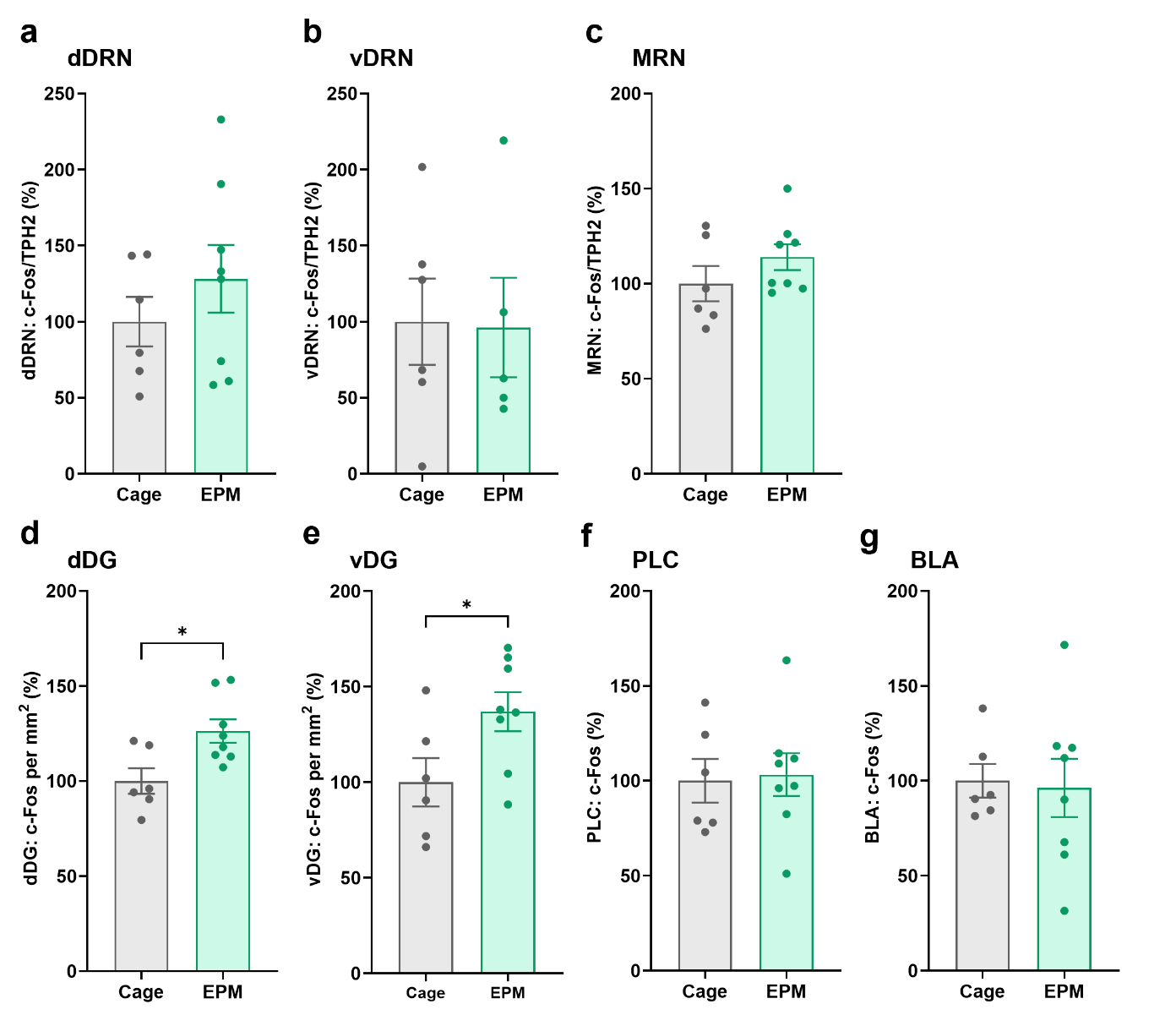


**Supplementary Figure 2 Effect of EPM exposure verus clean home cage on c-Fos immunoreactive neurons in anxiety-realted midbrain and forebrain regions**. Number of c-Fos/TPH2 double-labelled neurons in the (a) dorsal DRN (dDRN), (b) ventral DRN (vDRN) and (c) MRN (as a proportion of total number of TPH2 neurons). Number of c-Fos immunoreactive neurons in the (d) dentate gyrus of dorsal hippocampus (dDG) and (e) ventral hippocampus (vDG), (f) PLC and (g) BLA. Mean ± SEM values expressed as a % of home cage exposure group mean (home cage n=6, EPM n=5-8). Individual values are indicated by dots. Data analysed with Students t-test, * p<0.05.
